# Canal Construction Disrupts Camouflage in Two Sympatric Estuarine Crab Species

**DOI:** 10.1002/ece3.73820

**Published:** 2026-06-08

**Authors:** Yiran Huang, Sichen Lu, Fuxi Lai, Binyu Zhai, Yuhao Liao, Haitao Wang, Rongping Bu

**Affiliations:** ^1^ Pinglu Canal and Beibu Gulf Coastal Ecosystem Observation and Research Station of Guangxi, Guangxi Key Laboratory of Marine Environmental Disaster Processes and Ecological Protection Technology, College of Marine Sciences Beibu Gulf University Qinzhou China

**Keywords:** camouflage, canal construction, *Parasesarma pictum*, *Perisesarma bidens*

## Abstract

Estuarine ecosystems provide essential habitats for fiddler crabs, whose survival heavily relies on background‐matching camouflage. Anthropogenic modifications such as canal construction can alter the visual properties of these habitats, yet direct empirical evidence of its impact on crustacean camouflage remains limited. This study investigated the effects of habitat canalization on the camouflage effectiveness of two sympatric crab species, *Parasesarma pictum* and *Perisesarma bidens*. We quantified color matching using the chromatic just noticeable difference (CJND) metric, comparing individuals in engineered canal habitats versus adjacent natural mudflats. Results demonstrated a significant reduction in camouflage efficacy in canalized areas for both species. The CJND values were markedly higher in canal habitats ( *P. pictum* : 9.035(8.448); *P. bidens* : 12.410(6.730)) compared to natural habitats ( *P. pictum* : 6.505(4.753), Z = −7.696, *p* < 0.001; *P. bidens* : 8.625(5.658), Z = −11.678, *p* < 0.001), indicating a substantial decrease in body‐background color matching. Although the differences in luminance, pattern energy, and disruptive rate did not show the same trend, the weakening of color matching caused by the habitat alteration resulting from canal construction would also have a negative impact on the camouflage of the two crab species. These findings suggest that canalization alters substrate coloration and optical properties, thereby impairing the crabs' background‐matching camouflage. This sensory disruption may potentially increase predation risk and could represent an anthropogenic threat to benthic fauna. Our research offers an assessment of how human infrastructure might compromise adaptive camouflage, providing insights that may be useful for the conservation management of estuarine biodiversity.

## Graphical abstract

Estuarine ecosystems provide essential habitats for fiddler crabs, whose survival heavily relies on background‐matching camouflage. Anthropogenic modifications such as canal construction can alter the visual properties of these habitats, yet direct empirical evidence of their impact on crustacean camouflage remains limited. Our research provides a critical assessment of how human infrastructure compromises adaptive camouflage, offering vital insights for the conservation management of estuarine biodiversity.

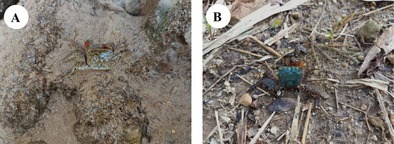

## 1 Introduction

Camouflage is a common anti‐predator strategy in which organisms minimize detection or recognition by resembling elements of their visual background (Endler 1978; Stevens and Merilaita 2009). Its efficacy is critical for both visually hunting predators and their prey (Cuthill et al. 2019). Common mechanisms include background matching—achieved through coloration and patterning that resemble the general surroundings—and disruptive coloration, which uses high‐contrast markings to break up the body's outline (Stevens and Merilaita 2011). Crucially, the success of any camouflage strategy is inherently context‐dependent, relying on a close match between the animal's appearance and the specific visual properties of its habitat.

Estuarine and intertidal crabs, often serving as bioindicators, provide a compelling model for studying context‐dependent camouflage. Their survival during foraging activities is closely tied to their ability to avoid visual predators, an effectiveness largely determined by microhabitat choice and consequent background‐matching (Price et al. 2019). Many crab species exhibit strong color‐environment associations, adapting to heterogeneous settings through habitat selection, physiological color change, or the attachment of decorative materials (Duarte et al. 2017; Stevens 2016). For instance, shore crabs ( *Carcinus maenas* ) and furrowed crabs (*Xantho hydrophilus*) actively select substrates that match their brightness, with the latter preferring complex, high‐contrast backgrounds, underscoring the role of behavioral plasticity in maintaining camouflage (Dyer and Stevens 2024; Twort and Stevens 2023). Similarly, juvenile land crabs (*Johngarthia lagostoma*) achieve higher concealment on sandy substrates via dark coloration, demonstrating active use of background‐matching strategies (João et al. 2022).

In the Anthropocene, however, human activities have become a dominant evolutionary force, imposing intense novel selection pressures on wildlife (Darimont et al. 2009; Palumbi 2001). Habitat modification often disrupts the visual environment, altering substrate color, texture, and complexity at a pace that can exceed the behavioral or physiological adaptive capacity of resident species (Heinrichs et al. 2016; Lai et al. 2025). This mismatch can render formerly effective camouflage strategies obsolete, reducing an individual's survival fitness (Coles et al. 2024). Some species may shift camouflage mechanisms in response; for example, the two spider crabs (*Schizophrys dahlak* and *Hyastenus hilgendorfi* ) in the modified Suez Canal environment have developed enhanced setae for debris attachment, adopting a passive decorative camouflage suited to turbid, unstable bottoms (Osman et al. 2021). Such observations suggest that anthropogenic habitat alteration can drive a transition from active background matching towards alternative camouflage strategies.

Canal construction represents a severe form of estuarine habitat modification, frequently simplifying structure, altering sediment composition, and changing optical properties. These alterations may critically impair benthic fauna like crabs that rely on precise visual background matching. Specifically, the *Parasesarma pictum* and the *P. bidens* are two ecologically important estuarine species whose camouflage is likely vulnerable to such changes. However, empirical evidence quantifying the impact of canalisation on the camouflage efficacy of these or similar species remains scarce.

**FIGURE 1:**
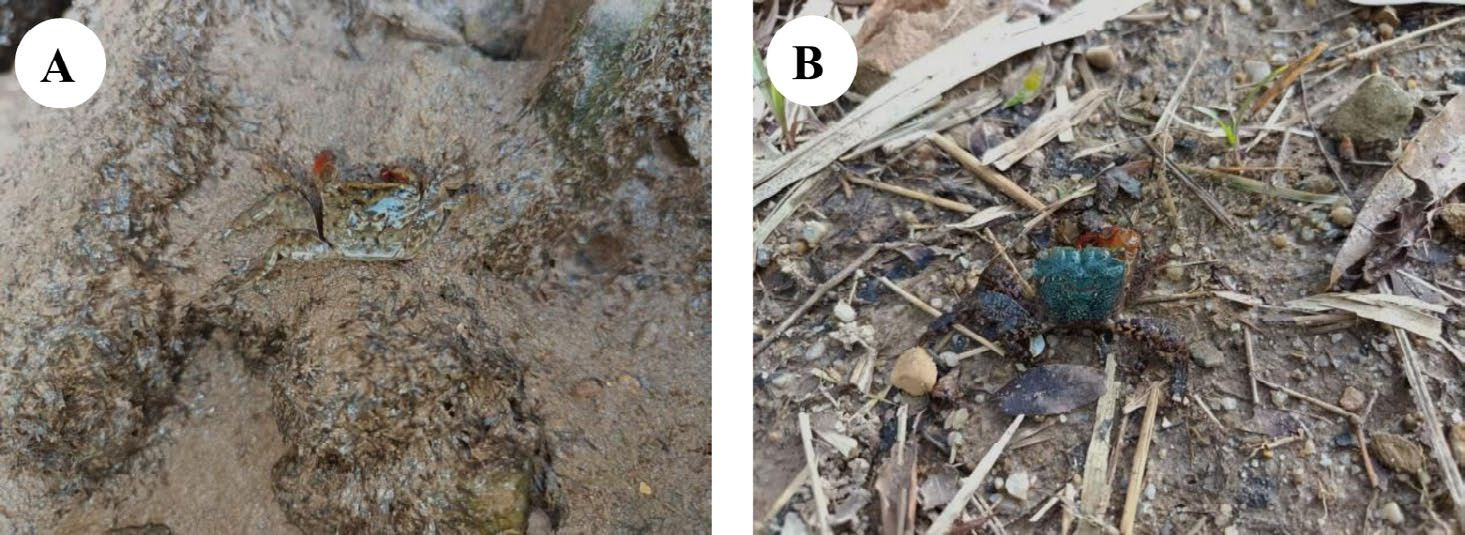
Photographs of *P. pictum* (A) *and P. bidens* (B).

Therefore, this study investigates the effect of canal construction on the camouflage function of two sympatric estuarine crabs. We systematically compared the background‐matching effectiveness of *P. pictum* (Figure 1A) and *P. bidens* (Figure 1B) in canalized versus adjacent natural habitats. This work aims to elucidate how human‐driven habitat alteration disrupts a key adaptive trait, deepening our understanding of anthropogenic impacts on benthic organism adaptability and providing insights relevant for conservation in increasingly modified ecosystems.

## 2 Materials and Methods

### 2.1 Sampling

**FIGURE 2:**
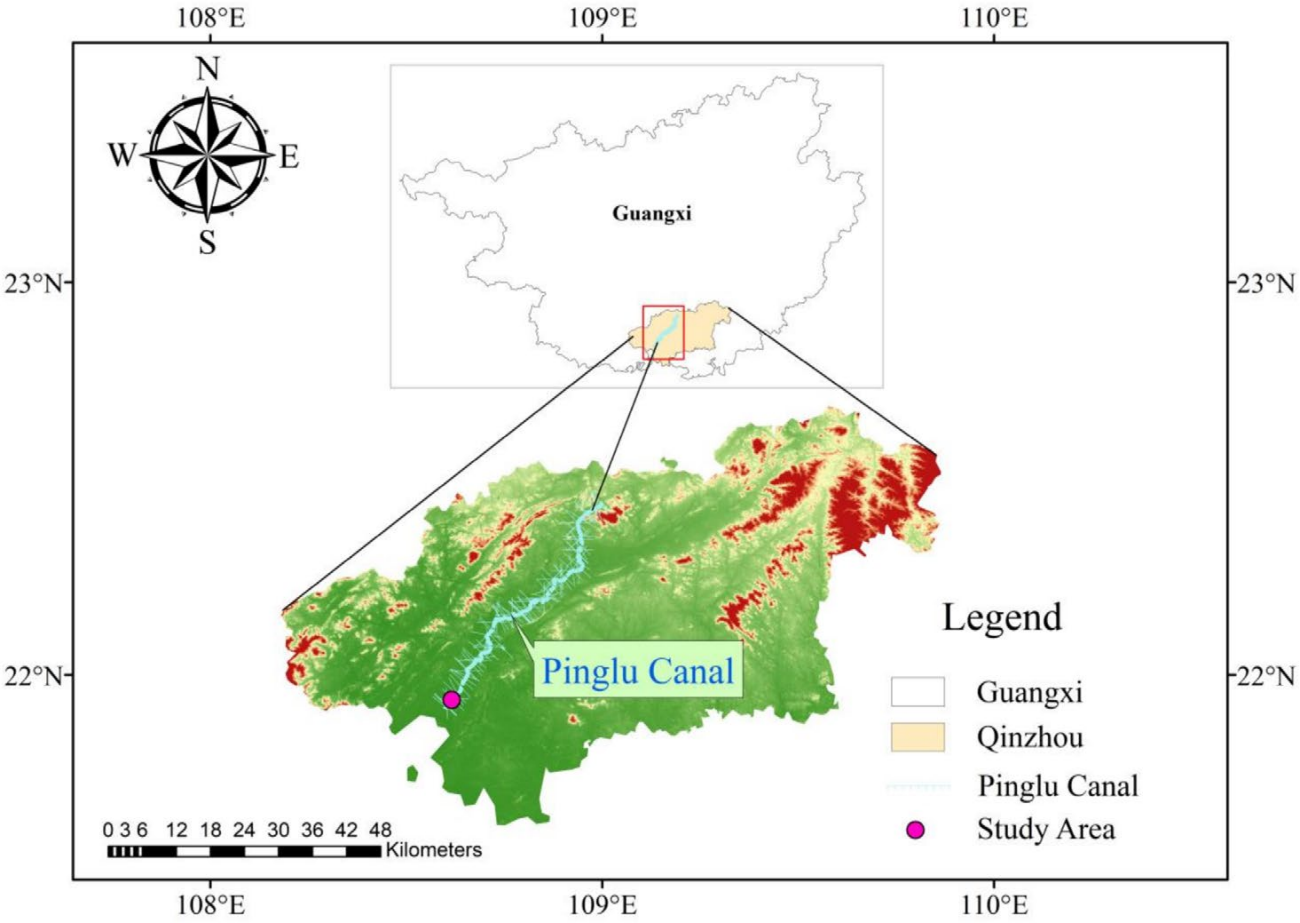
Study area map.

The Pinglu Canal, a landmark project designed to connect inland rivers with the sea. As a cornerstone of the Western Land‐Sea New Corridor and a pivotal initiative in building China into a leading transportation nation, it holds strategic importance for promoting the development of Guangxi and the broader southwestern region. Construction began in August 2022 at the estuary of the Pingtang River in the Xijin Reservoir, Hengzhou City, Nanning. The canal extends through Luwu Town in Lingshan County, Qinzhou, follows the Qin River, and finally enters the Beibu Gulf, with a total planned length of approximately 134.2 km. The project involves extensive widening, dredging, and straightening of the main and tributary channels of the Qinjiang River. These modifications alter the river's planar and longitudinal morphology, thereby changing the basin's original hydrological regime. As a result, water clarity has decreased, and the riverbed substrate has become more homogeneous. Our sampling sites are situated along the Pinglu Canal, at approximately 21.9° N, 108.6° E (Figure 2).

### 2.2 Image Target Acquisition and Calibration

To analyze the camouflage effectiveness of crabs against the substrate background (approx. 100 cm × 80 cm), a black sunshade was used during sunny daytime hours (09:00–16:00) to block direct sunlight and ensure uniform illumination. Multispectral imaging was performed with a Nikon D7000 camera equipped with a 105 mm lens. Visible light images were captured through a Baader UV/IR Cut filter (transmittance: 400–700 nm), and ultraviolet (UV) images through a Baader U filter (transmittance: 300–400 nm). The combined system response defined four image channels—Ultraviolet (UV: 300–400 nm), Shortwave (SW: 400–550 nm), Midwave (MW: 420–620 nm), and Longwave (LW: 560–700 nm)—corresponding to the broad visual spectrum of avian predators.

During each capture, crab specimens were positioned together with calibrated reflectance standard cards (10% and 80% reflectance, calibration range 300–750 nm) at the center of the image. White balance was adjusted using these standards prior to imaging to minimize color bias. All images were saved in uncompressed RAW format with consistent camera settings, and the camera was mounted on a tripod to maintain the optical axis perpendicular to the sample plane.

To correct for potential inter‐image illumination variation, the “Generate multispectral image” function in ImageJ 1.53e (Troscianko and Stevens 2015) was applied. Linear normalization based on the 10% and 80% reflectance regions of the standard cards (including UV images) yielded calibrated multispectral images.

A total of 14 specimens of *P. pictum* and 21 specimens of *P. bidens* were collected from canal habitats. There is no significant difference in body coloration between crabs from the canal habitat and those from the natural habitat. Photographs were taken of 21 natural habitats and 49 canal habitats. Each crab was photographed against the same background. After linearization correction, the crabs were then added to different habitat backgrounds using software for analysis. All images were linearized and illumination‐corrected using ImageJ 1.53e (Wayne Rasband and contributors, National Institutes of Health, USA). Lower color difference values indicate higher color matching between crab and substrate (Twort and Stevens 2023). For each habitat, five locations were randomly selected. The differences between the crab and the habitat at each of the five locations were calculated separately, and then the average was taken. All image analysis was performed by a third party who was unaware of the research intent. The ROIs were manually added by the personnel involved in the image analysis.

### 2.3 Color Quantification

For the calibrated images, we quantified the brightness, chromaticity, and pattern energy differences between the crab and its substrate using the image analysis functions in ImageJ 1.53e. Specifically, we calculated the just‐noticeable difference (JND) for chromaticity and luminance, as well as the pattern energy difference (PED), following established methods (Vorobyev and Osorio 1998; Troscianko and Stevens 2015; van den Berg et al. 2020).

Given that many avian predators are tetrachromatic, utilizing four cone types sensitive to long‐wave (LW), medium‐wave (MW), short‐wave (SW), and ultraviolet (UV) light (Cuthill et al. 2000), we employed an avian visual model (Blue Tit, *Cyanistes caeruleus* ) to evaluate chromatic differences. Within this framework, a lower JND value indicates a reduced perceptual difference and, thus, higher camouflage effectiveness.

Using a custom analysis toolkit, images were converted to cone‐catch format under standard daylight illumination (D65) with a Weber fraction of 0.02. We then analyzed the chromaticity, luminance, and pattern energy within defined regions of interest (Troscianko and Stevens 2015). For chromatic and luminance JND calculations, pixel sampling was performed across a scale range from 2 to 512 pixels, with scale size increasing exponentially by a factor of 1.414 at each step. Luminance values were scaled from 0 to 65,535, corresponding to the full dynamic range of a 32‐bit TIFF image (Nokelainen et al. 2020).

This methodological framework enables a comparative assessment of the background‐matching effectiveness for two fiddler crab species between natural and canal‐modified habitats. The analysis aims to elucidate the dependence of effective camouflage on intact habitat conditions.

### 2.4 GabRat Determination

The corrected image was converted into a binary mask. A Gabor filter was then applied at multiple orientations to each pixel along the prey's edge, comparing the response in the target region with that at the corresponding pixel on the prey's true contour (Gabor 1946; Troscianko et al. 2017). Gabor wavelets are sensitive to image edges and provide strong selectivity for both orientation and spatial scale. They are also robust to variation in lighting conditions, ensuring reliable performance under uneven illumination (Gabor 1946). For each point along the prey's outline, an edge disruption ratio was computed by dividing the filter response at the computer‐detected edge by the sum of the responses at the detected edge and the true prey edge. The mean of these ratios across the entire outline was taken as the overall edge disruption metric (GabRat).

GabRat quantifies the proportion of spurious edges to true edges along the outline of the crab carapace, indicating the degree to which internal patterning disrupts the perception of the true body contour. Values range from 0 to 1, where higher GabRat values reflect stronger edge disruption—meaning the true outline is better concealed within the background, indicative of effective disruptive camouflage. Conversely, lower values correspond to more visible edges and poorer disruptive concealment. Following Troscianko et al. (2017), GabRat values above 0.2 were considered to indicate some disruptive effect, and values above 0.4 were classified as high disruptive concealment.

In this study, GabRat was measured using the corresponding tool in ImageJ 1.53e (Troscianko and Stevens 2015; Troscianko et al. 2017). The procedure consisted of the following steps: (1) conversion of the corrected image into a binary mask; (2) manual selection of the true carapace edge; (3) configuration of filter parameters and measurement angles based on the image pixel‐to‐scale ratio; (4) GabRat measurement at five randomly selected positions of the crab against the substrate; and (5) calculation of the mean value as the GabRat for that substrate type. This approach enabled a comparative analysis of disruptive coloration between the two sesarmid crab species in natural versus canal‐modified habitats, thereby assessing the role of habitat integrity in the efficacy of disruptive camouflage.

### 2.5 Scanning Electron Microscopy of Crab Shell

To investigate whether the setae on the surfaces of two crab species can serve as attachment substrates for environmental materials for camouflage purposes, we collected crabs with carapace widths ranging from 12 to 45 mm. After air‐drying, the specimens were subjected to scanning electron microscopy (SEM).

### 2.6 Statistical Analysis

Statistical analysis of CJND, LJND, PED, and GabRat values for the spotted crab and the double‐toothed crab across different habitats was performed using OriginPro software. A Kolmogorov–Smirnov test was conducted to assess normality prior to analysis. Data conforming to a normal distribution are presented as mean ± SE, while data not conforming to a normal distribution are presented as medians and interquartile ranges (IQR). If the data conform to a normal distribution, an independent samples *t*‐test was used; if not, the nonparametric Mann–Whitney *U* test was applied. The significance level was set at *p* < 0.05.

## 3 Results

### 3.1 SEM Micrograph of Setae

**FIGURE 3:**
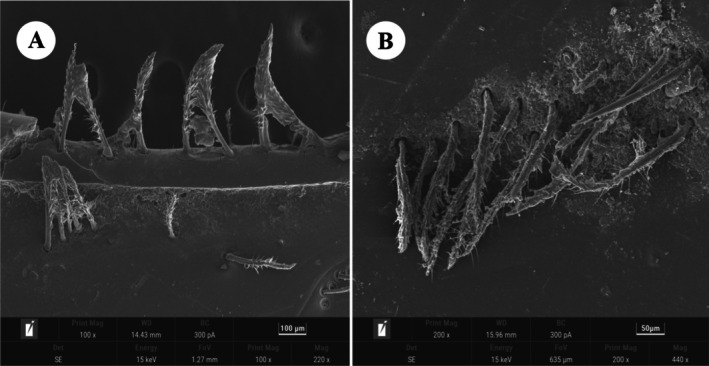
The setae of the *P. pictum* exhibit a brush‐like structure with distinct branching. Numerous minute lateral cilia are symmetrically distributed on both sides of the main axis (A). The setae of the *P. bidens* also display a typical brush‐like morphology, featuring symmetrical branching but lacking hook‐like or conical spines.

The setae of the *P. pictum* (Figure 3A) and the *P. bidens* (Figure 3B) primarily exhibit a brush‐like structure. These setae are widely distributed across the palmar surface of the chelae, the carpus, the anterior margins of the walking legs, and the perioral region. They feature a distinct central axis with lateral branches, offering a large surface area with dense arrangement and numerous branches or cilia. Collectively, they resemble a soft brush.

### 3.2 Comparison Results of Multiple Indicators

**FIGURE 4:**
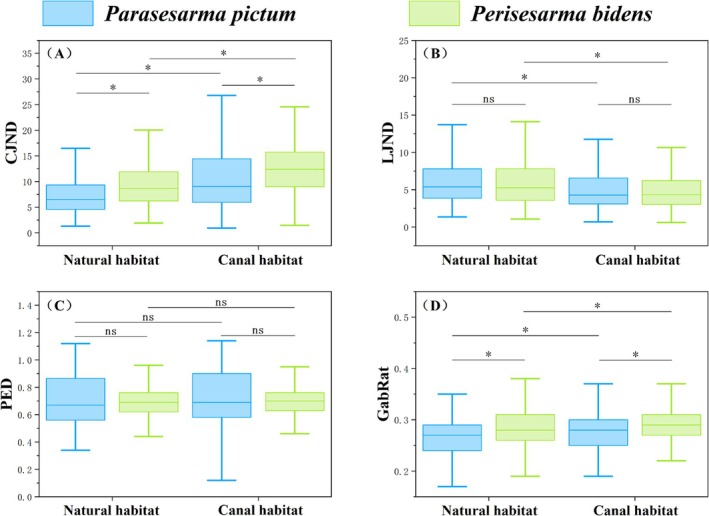
The CJND (A), LJND (B), PED (C), and GabRat (D) indices between the *P. pictum* and the *P. bidens* in different habitats (natural habitat and canal habitat). “*” indicates significant differences between the two, while “ns” indicates no significant differences.

In adjacent natural habitats, the CJND of the *P. pictum* 6.505(4.753) was significantly smaller than the *P. bidens* (8.625(5.658), Z = −7.064, *p* < 0.001); in canal habitats, the CJND of the *P. pictum* 9.035(8.448) was also significantly smaller than the *P. bidens* (12.410(6.730), Z = −9.742, *p* < 0.001). The CJND values for both species of crab in canal habitats ( *P. pictum* : 9.035(8.448); *P. bidens* : 12.410(6.730)) were significantly higher than those in natural habitats ( *P. pictum* : 6.505(4.753), Z = −7.696, *p* < 0.001; *P. bidens* : 8.625(5.658), Z = −11.678, *p* < 0.001) (Figure 4A).

In adjacent natural habitats and canal habitats, no significant differences were observed in the LJND values between the *P. pictum* (Natural habitat: 5.365(3.930); Canal habitat: 4.280(3.465)) and the *P. bidens* (Natural habitat: 5.260(4.235), Z = −0.793, *p* = 0.428; Canal habitat: 4.330(3.170), Z = −0.693, *p* = 0.488). The LJND values of both species of crab in canal habitats ( *P. pictum* : 4.280(3.465); *P. bidens* : 4.330(3.170)) were significantly lower than those in adjacent natural habitats ( *P. pictum* : 5.365(3.930), Z = −5.753, *p* < 0.001; *P. bidens* : 5.260(4.235), Z = −6.495, *p* < 0.001) (Figure 4B).

In adjacent natural habitats and canal habitats, no significant differences were observed in the PED values between the *P. pictum* (Natural habitat: 0.670(0.303); Canal habitat: 0.690(0.318)) and the *P. bidens* (Natural habitat: 0.690(0.140), *Z* = −0.438, *p* = 0.662; Canal habitat: 0.700(0.130), Z = −0.618, *p* = 0.537). There were no significant differences in PED values between the two species of crab in canal habitats and adjacent natural habitats ( *P. pictum* : 0.690(0.318); 0.670(0.303), Z = −1.696, *p* = 0.090; *P. bidens* : 0.700(0.130); 0.690(0.140), Z = −1.391, *p* = 0.164) (Figure 4C).

Whether in adjacent natural habitats or canal habitats, the GabRat values for the *P. pictum* (Natural habitat: 0.270(0.050); Canal habitat: 0.280(0.050)) and the *P. bidens* (Natural habitat: 0.280(0.050), Z = −5.024, *P* < 0.001; Canal habitat: 0.290(0.040), Z = −7.995, *P* < 0.001) exhibit significant differences. The GabRat values of the *P. pictum* were significantly higher in canal habitats 0.280(0.050) than in adjacent natural habitats (0.270(0.050), Z = −3.287, *p* = 0.001), indicating that them may partially compensate for reduced color matching by enhancing cryptic efficiency. The *P. bidens* exhibited a similar trend, with its GabRat values also significantly higher in canal habitats 0.290(040) compared to adjacent natural habitats (0.280(0.050), Z = −3.185, *p* = 0.001) (Figure 4D).

## 4 Discussion

Our results suggest that the CJND for both crab species tended to be lower in natural habitats than in canal habitats. A lower CJND may indicate a reduced perceptual color difference between the individual and its background from an avian predator's perspective, potentially enhancing concealment through improved background matching (Nokelainen et al. 2017; Duarte et al. 2021). Canal construction, by altering substrate structure and ambient light conditions, might impair the crabs' ability to achieve chromatic concordance with their environment. This possible degradation in background‐matching efficacy could increase predation risk (Stevens and Merilaita 2011; Coles et al. 2024), potentially disrupting a long‐evolved camouflage strategy and exposing these benthic crabs to heightened predatory pressure.

Notably, a significant interspecific difference in color‐matching capability was observed, attributable to their distinct inherent colorations. This aligns with studies on how color polymorphism within sympatric populations can differentially influence predator–prey dynamics (Russell and Dierssen 2015). Consequently, the environmental changes wrought by canalization may impose a higher relative predation risk on *P. bidens* , whereas *P. pictum* , with its superior inherent color‐matching ability, appears better equipped to maintain effective camouflage and a survival advantage in disturbed settings.

Contrasting with chromatic results, the luminance JND (LJND) for both species was lower in the canal habitat, indicating a significant reduction in brightness contrast with the substrate. This suggests that canal modifications have homogenized the optical properties of the substrate, possibly through increased suspension of fine sediments. Jung et al. (2024) noted that fine particles enhance light scattering, elevating overall surface reflectance and reducing local brightness variation, thereby creating a more uniform visual background. The consequent reduction in brightness contrast between crab and substrate in canals may enhance brightness matching, potentially compensating for poorer chromatic camouflage. The lack of significant difference in PED, despite chromatic disparities, may stem from this shift towards brightness adaptation in *P. bidens* , while *P. pictum* maintains a balance between color and luminance for more stable pattern matching.

Both *P. pictum* and *P. bidens* possess carapaces covered with brush‐like setae capable of trapping environmental particles. This morphological trait provides a mechanism for rapid, plastic adaptation via decorative camouflage—a strategy documented in other crustaceans. For instance, the *Notomithrax ursus* actively arranges seaweed for individualized camouflage (McLay 2020), and the two spider crabs (*Schizophrys dahlak* and *Hyastenus hilgendorfi* ) in the Suez Canal develop complex setae to enhance debris attachment for passive camouflage in turbid environments (Osman et al. 2021). Similarly, pappose setae in *Libinia* spp. trap particulates to improve background matching in color and texture (Wortham 2021). Our observations suggest that habitat alteration may drive a strategic shift from reliance on active physiological color change towards increased use of such decorative or generalist camouflage mechanisms. In our study, we did not deliberately remove the attachments on the crabs, yet their camouflage effectiveness still diminished. It appears that their ornamental camouflage abilities may not be sufficient to counteract the decline in camouflage effectiveness caused by environmental changes.

Regarding disruptive coloration, GabRat values for *P. bidens* were consistently higher than for *P. pictum* across habitats, indicating a stable interspecific difference in edge disruption. More importantly, GabRat increased significantly in the canal habitat for both species, implying that anthropogenic modification heightens their reliance on disruptive camouflage. However, as values remained below the 0.4 threshold associated with high disruptive concealment (Troscianko et al. 2017), this strategy does not appear to be their primary anti‐predator defense. The “camouflage threshold theory” posits that even suboptimal camouflage can generate visual noise and reduce detection probability (Rodríguez‐Gironés and Maldonado 2020). The spotted patterning on *P. pictum* , while not forming high‐contrast disruptive patches, may function as a weak disruptive pattern (Troscianko et al. 2021), contributing to overall concealment alongside other strategies.

## 5 Conclusion

This study compared the camouflage efficacy of two estuarine crab species, *P. pictum* and *P. bidens* , between canal‐modified areas and adjacent natural habitats. Our findings suggest that human‐induced habitat alteration, specifically canal construction, may impair the background‐matching camouflage of these benthic crustaceans to some extent. Although both species possess setae that aid in passive background assimilation, their camouflage effectiveness was substantially reduced in homogenized canal substrates. The simplification of substrate color and texture, along with altered optical conditions, directly compromises their primary anti‐predator strategy. These changes are likely to increase predation pressure, alter interspecific dynamics, and may drive shifts in the functional structure of estuarine ecosystems. This work underscores that maintaining substrate heterogeneity is critical in coastal engineering and restoration projects to preserve the adaptive camouflage of resident fauna and broader biodiversity. However, our study used the visual model of the blue tit to evaluate the camouflage effectiveness of crabs, which may deviate to some extent from that of actual avian predators. Future research could construct visual models of their real predators to conduct further studies.

## Author Contributions

**Yiran Huang:** data curation (lead), investigation (lead), writing – original draft (lead), writing – review and editing (supporting). **Sichen Lu:** data curation (lead), investigation (lead), writing – original draft (lead). **Fuxi Lai:** data curation (supporting), investigation (supporting). **Binyu Zhai:** data curation (supporting), investigation (supporting). **Yuhao Liao:** data curation (lead). **Haitao Wang:** data curation (supporting). **Rongping Bu:** funding acquisition (lead), methodology (lead), project administration (lead), resources (lead), supervision (supporting), writing – original draft (supporting), writing – review and editing (lead).

## Funding

This work was supported by the Natural Science Foundation of Guangxi, China (No. 2024GXNSFBA010371) and University Students’ Innovation and Entrepreneurship Training Program (S202411607019 and S202511607120).

## Disclosure

*Institutional Review Board statement*: Fieldwork was carried out in strict accordance with the guidelines of the Animal Research Ethics Committee of Beibu Gulf University, which conforms to the laws of China.

## Conflicts of Interest

The authors declare no conflicts of interest.

## Data Availability

I confirm that the Data Availability Statement is included in the main file of my submission, and that access to all necessary data files is provided to editors and reviewers.“Data Available Upon Request” is unacceptable. The data is requested for the peer review process. Dryad at: 10.5061/dryad.08kprr5h2.
